# Antimicrobial Resistance and Pathotypes of *Escherichia coli* Isolates from Yellow-Legged Seagulls (*Larus michahellis*) in Central Italy

**DOI:** 10.3390/ani14213048

**Published:** 2024-10-22

**Authors:** Giulia Cagnoli, Fabrizio Bertelloni, Renato Ceccherelli, Valentina Virginia Ebani

**Affiliations:** 1 Department of Veterinary Sciences, University of Pisa, 56124 Pisa, Italy; giulia.cagnoli@phd.unipi.it (G.C.); fabrizio.bertelloni@unipi.it (F.B.); 2 CRUMA-LIPU, 57121 Livorno, Italy; apusvet.cruma@libero.it; 3 Centre for Climate Change Impact, University of Pisa, 56124 Pisa, Italy

**Keywords:** *Escherichia coli*, seagulls, pathotypes, necrotoxigenic *E. coli* (NTEC), antimicrobial resistance

## Abstract

Seagulls are synanthropic wild birds that can contaminate, through their droppings, beaches, urban and peri-urban environments. This concern is more serious when seagulls eliminate antimicrobial-resistant pathogenic bacteria. This study analyzed the fecal samples from 137 yellow-legged seagulls (*Larus michahellis*) from Central Italy. A total of 218 *Escherichia coli* strains were isolated and analyzed for phenotypic and genotypic antimicrobial resistance and to identify the virulence genes characterizing different pathotypes. The disk diffusion method on all isolates found relevant resistance rates to ampicillin (38.99%), tetracycline (23.85%), and enrofloxacin (21.10%). On the basis of all results obtained with this test, 62 (28.44%) isolates were classified as multidrug-resistant (MDR) and 6 (2.75%) as extensive drug-resistant (XDR). Molecular analyses conducted on the strains phenotypically resistant to carbapenems, cephalosporins, and penicillins found 9/37 (24.32%) strains positive for *bla*_OXA-48_, 52/103 (50.49%) for *bla*_TEM,_ 12/103 (11.65%) for *bla*_CMY2_, 3/103 (2.91%) for *bla*_CTX_, and 1/103 (0.97%,) for *bla*_SHV_. PCR to detect virulence genes characterizing different pathotypes found that 40 (18.35%) isolates had the *ast*A gene, indicative of the enteroaggregative (EAEC) pathotype, 2 (0.92%) had *cnf*1, 2 (0.92%) had *cnf*2, and 1 (0.46%) had *cdt*-IV. All five (2.29%) strains were reportable as necrotoxigenic (NTEC), while 4 (1.83%) had both *eae*A and *esc*V, reportable as enteropathogenic (EPEC). Measures to limit seagulls’ access where humans and other animals reside are pivotal to reduce the risk of infection with antimicrobial-resistant and pathogenetic *E. coli* strains.

## Simple Summary

*Escherichia coli* consists of different pathotypes, such as enteropathogenic (EPEC), enterotoxigenic (ETEC), enteroaggregative (EAEC), shiga-toxin (STEC), enterohaemorrhagic (EHEC), and necrotoxigenic (NTEC). They are responsible for severe human clinical forms that often are difficult to treat because of the resistance to one or more antimicrobials. Animals, including birds, may be involved in the epidemiology of *E. coli* antimicrobial-resistant pathotypes acting as reservoirs. Seagulls are synanthropic wild birds largely present not only along the coastal areas, but also in hinterlands where they can contaminate numerous environments, including urban and farm areas, through their droppings. Monitoring of seagulls is a useful tool to obtain information about the circulation of pathogenic bacteria and to verify the antimicrobial resistance trend.

## 1. Introduction

*Escherichia coli* is an opportunistic Gram-negative bacterium, belonging to the family Enterobacteriaceae, commensal of the human and animal intestinal tract. This species encompasses several pathotypes responsible for intestinal and extra-intestinal infections. Urinary and genital tract infections, meningitis, and septicemia, are the most frequent extra-intestinal forms encountered in animals and humans [1]. Different *E. coli* diarrhoeagenic pathotypes are involved in the enteric forms. They act with different mechanisms in relation to their virulence traits.

Enteropathogenic *E. coli* (EPEC) strains produce the adherence factor intimin; it is encoded by the *eae* gene and allows bacteria to adhere to enterocytes causing microvilli loss and consequent diarrhea [2]. Enterotoxigenic *E. coli* (ETEC) strains are characterized by two groups of virulence factors. The first group includes heat-stable (ST) and heat-labile enterotoxins (LT) [3]. The second group includes colonization factors, such as fimbriae, that help the bacteria to adhere to the ileum [3]. All enteroaggregative *E. coli* (EAEC) strains are characterized by their aggregative-adherence pattern, designated the stacked-brick configuration, which is mostly mediated by aggregative-adherence fimbriae (AAF) [4]. Most EAEC bacteria harbor additional virulence factors such as the EAEC heat-stable enterotoxin and serine proteases [4]. Shiga toxin-producing *E. coli* (STEC) are strains producing two of the most potent bacterial toxins: Stx1, homologous to the Stx produced by *Shigella dysenteriae* type 1, and Stx2, which includes several subtypes. These toxins are toxic to colonic, ileal epithelial, and endothelial cells [5,6,7]. Enterohemorrhagic *E. coli* (EHEC) are STEC strains having intimin and hemolysin as additional virulence factors. Hemolysin, encoded by the *hly* gene, contributes to the pathogenesis by different mechanisms such as hemolysis, induction of pro-inflammatory reactions, and epithelial and endothelial cells damage [8]. STEC and EHEC cause different clinical manifestations in humans, including the asymptomatic form, bloody or severe diarrhea and systemic diseases, such as hemorrhagic colitis (HC), and the life-threatening hemolytic–uremic syndrome (HUS), which is the main cause of acute renal failure in children [9]. Necrotoxigenic *E. coli* (NTEC) have different virulence factors, including fimbrial and afimbrial adhesins, siderophores, and toxins. The cytolethal distending toxin (Cdt) impairs host defense by the holding cell cycle and by apoptosis in epithelial cells and lymphocytes, and subsequent impairing of acquired immunity [10]; it can also alter macrophage function leading to a pro-inflammatory response [10,11]. In addition, NTEC strains have the two cytotoxic necrotizing factors CNF1 and CNF2, which induce multi-nucleation and necrosis of eukaryotic cells [11].

Antimicrobial resistance is a major global challenge affecting animals and humans [12]. Most *E. coli* strains involved in infections of mammals and birds are resistant to several antimicrobials [13].

Wild birds often harbor pathogens, including antimicrobial-resistant bacteria, which can disseminate in the environment through their feces [14,15,16,17,18]. Seagulls are synanthropic wild birds largely present not only along the coastal areas, but also in hinterlands. They nest on private houses, hotels, large warehouses, and shipyards. They can fly great distances for food, often to landfill sites, especially during the winter, and sewage outlets or agricultural land and farm areas [19]. In addition, they have been proven to transport bacteria from human and animal waste to recreational beaches [19].

Previous studies investigated *E. coli* populations in wild birds, including seagulls [15,18,20,21,22]. Wild avifauna has been considered a possible bioindicator of antibiotic resistance; therefore, studies have been focused on the determination of the antimicrobial resistance patterns of *E. coli*. Conversely, to the best of our knowledge, this is the first survey aimed to investigate the role of seagulls present in Italy in the dissemination of antimicrobial-resistant *E. coli*, as well as of different *E. coli* pathotypes.

In fact, the aim of the present study was to investigate the occurrence of *E. coli* strains belonging to different pathotypes in fecal samples collected from yellow-legged seagulls (*Larus michahellis*) recovered in a rescue center in Central Italy and to study their phenotypic and genotypic characters of antimicrobial resistance.

## 2. Materials and Methods

### 2.1. Sampling

During the summer seasons of 2022 and 2023, fecal samples were collected from 137 yellow-legged seagulls (*Larus michahellis*) recovered at a wildlife rescue center in Central Italy. The gulls were recovering from trauma and kept in single cages; feces were sampled from the bottom of each cage to avoid animals’ stress. Samples were collected as soon as possible after the animal’s arrival at the rescue center, usually within 48 h. Only gulls not yet receiving antibiotic treatments were enrolled in the study. No ethical approval was required because no biological materials were sampled directly from the birds. Each fecal sample was collected in a sterile plastic tube and transferred within 3 h, kept in a cool bag at 4 °C, and sent to the Avian Pathology Laboratories of the Department of Veterinary Sciences, University of Pisa, where it was immediately submitted to bacteriological analyses.

### 2.2. Escherichia coli Isolation

A swab from each fecal sample was pre-enriched in buffered peptone water (BPW) (Oxoid Ltd., Basingstoke, UK) at 37 °C for 24 h; successively, a loop was streaked onto selective Tryptone Bile X-GLUC (TBX) agar (Biolife, Milan, Italy) and incubated at 42 °C for 24 h. From each sample, 2 distinct colonies were collected, if possible, streaked on Tryptic Soy Agar (TSA) (Biolife).

*Escherichia coli* isolated strains were stored, until needed for further analyses, in Brain–Heart Infusion (BHI) broth (Oxoid Ltd.), with the addition of 30% glycerol as a cryoprotectant, at −80 °C.

### 2.3. Antimicrobial Susceptibility Tests

All *E. coli* isolates were analyzed for antimicrobial resistance using the Kirby–Bauer disk diffusion test, following CLSI guidelines [23].

A total of 15 different antibiotic molecules, classified into 9 classes, were tested. The following antimicrobial disks (Oxoid, Ltd.) were employed: ampicillin (10 µg), amoxicillin-clavulanate (20/10 µg), cefoxitin (30 µg), cefotaxime (30 µg), ceftiofur (30 µg), imipenem (10 µg), ertapenem (10 µg), aztreonam (30 µg), chloramphenicol (30 µg), tetracycline (30 µg), enrofloxacin (5 µg), ciprofloxacin (5 µg), gentamicin (10 µg), amikacin (30 µg), and trimethoprim-sulfamethoxazole (1.25–23.75 µg).

*Escherichia coli* ATCC25922 was included as control. The obtained inhibition zones were interpreted according to CLSI [24].

The investigated strains were classified as multidrug-resistant (MDR), extensively drug-resistant (XDR), or pandrug-resistant (PDR) on the basis of the phenotypic resistance results. Briefly, MDR is defined as non-susceptibility to at least one agent in three or more antimicrobial categories. XDR is defined as non-susceptibility to at least one agent in all but two or fewer antimicrobial categories. PDR is defined as non-susceptibility to all agents in all antimicrobial categories [25].

### 2.4. Molecular Analyses

DNA was extracted from fresh *E. coli* strains, cultured on TSA, employing a commercial kit, Quick-DNA Miniprep Plus Kit (Zymo Research, Irvine, CA, USA), following the manufacturer’s instructions.

All PCR assays described below were performed in the automated thermocycler SimpliAmp™ Thermal Cycler, (Applied Biosystems, Waltham, MA, USA). In all PCR assays, sterile water instead of DNA was included as a negative control, whereas DNA from previously isolated and characterized *E. coli* strains, selected in relation to the searched gene, was added as positive control. All PCR products were analyzed by electrophoresis on 1.5% agarose gel at 100 V for 45 min, using 100 bp DNA Ladder Ready to Load (Solis BioDyne, Tartu, Estonia) as a DNA marker; the gel was stained with ethidium bromide and observed under UV light.

Positive PCR products were submitted to sequencing analyses (BMR Genomics, Padova, Italy). The obtained sequences were analyzed using BioEdit and compared with online gene bank databases: Basic Local Alignment Search Tool (BLAST) and FASTA (https://www.ebi.ac.uk/Tools/sss/fasta/) (Accessed on 10 April 2024).

#### 2.4.1. Identification of *E. coli* Strains

Pure isolates were confirmed as *E. coli* by the use of a species-specific PCR, with the primers uspAF (5′-CCGATACGCTGCCAATCAGT-3′) and uspAR (5′-ACGCAGACCGTAGGCCAGAT-3′), which allow the amplification of a 884 bp fragment of the *uspA* gene; PCR conditions consisted of 30 cycles, each of 94 °C for 2 min, 70 °C for 1 min, and 72 °C for 1 min [26].

#### 2.4.2. Genotypic Resistance

The isolates showing phenotypic resistance to penicillins (ampicillin and/or amoxicillin-clavulanate) and/or cephalosporins (cefoxitin, cefotaxime and/or ceftiofur) were submitted to molecular analyses to investigate the presence of genes *bla*_TEM_, *bla*_SHV_, and *bla*_CTX-M_, coding for extended spectrum β-lactamases (ESBL). The same strains were also tested for the presence of *bla*_CMY1_ and *bla*_CMY2_ genes coding for AmpC β-lactamases. Furthermore, strains phenotypically resistant to carbapenems (imipenem and/or ertapenem) were tested for the presence of *bla*_NDM_, *bla*_VIM_, *bla*_IMP_, *bla*_KPC_, and *bla*_OXA-48_ genes, coding for carbapenemases. Primers and PCR conditions were reported in Table 1.

#### 2.4.3. Virulence Factors

The selected *E. coli* strains were tested for the presence of 20 virulence genes, belonging to 7 pathotypes: STEC, EHEC, EPEC, ETEC, EAEC, EIEC, and NTEC (Table 2). Specifically, these were *stx1* and *stx2*, characteristics of the STEC pathotype, and the *hlyA* gene specific of EHEC; *eae*A gene that is common to both EHEC and EPEC; *esc*V, *bfp*B, and *ent* that characterize EPEC; *elt*, *est*Ia, and *est*Ib for ETEC; *ast*A, *agg*R, and *pic* for EAEC; *inv*E for EIEC; and *cnf*1, *cnf*2, *cdt*-I, *cdt*-II, *cdt*-III, and *cdt*-IV for NTEC.

## 3. Results

### 3.1. Escherichia coli Isolation

*E. coli* strains were isolated from 110 (80.29%, CI: 73.63–86.95%) of the 137 analyzed fecal samples; of these *E. coli*-positive samples, 2 yielded one isolate for each while the remaining 108 yielded two strains, making a total of 218 isolates.

### 3.2. Agar Disk Diffusion Method

**Figure 1:**
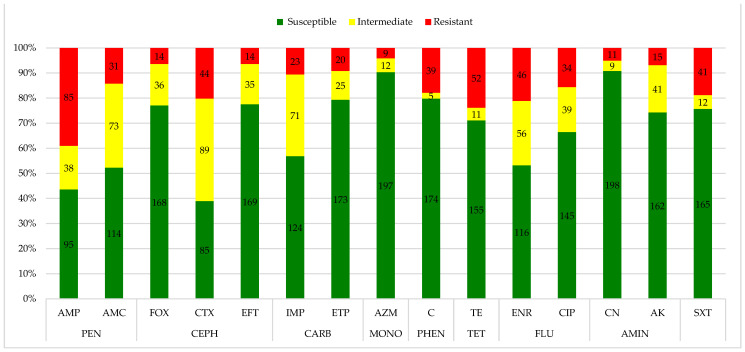
Antimicrobial resistance profile of *Escherichia coli* isolates (n.218) from seagulls. Legend: AMP: ampicillin; AMC: amoxicillin-clavulanate; FOX: cefoxitin; CTX: cefotaxime; EFT: ceftiofur; IMP: imipenem; ETP: ertapenem; AZM: aztreonam; C: chloramphenicol; TE: tetracycline; ENR: enrofloxacin; CIP: ciprofloxacin; CN: gentamicin; AK: amikacin; SXT: trimethoprim-sulfamethoxazole; PEN: penicillins; CEPH: cephalosporins; CARB: carbapenems; MONO: monobactams; PHEN: phenicols; TET: tetracyclines; FLU: fluoroquinolones; AMIN: aminoglycosides.

The disk diffusion method on the 218 isolates found relevant resistance rates to ampicillin (38.99%; 95% CI: 33.49–46.49%), tetracycline (23.85%; 95% CI: 18.19–29.51%), and enrofloxacin (21.10%; 95% CI: 15.68–26.52%). The most prevalent susceptibility was observed for gentamicin (90.83%; 95% CI: 87.00–94.66%), aztreonam (90.37%; 95% CI: 86.45–94.29), chloramphenicol (79.82% 95% CI: 74.49–85.15%), ertapenem (79.36%; 95% CI: 73.99–84.73%), ceftiofur (77.52%; 95% CI: 71.98–83.06%), and cefoxitin (77.06%; 95% CI: 71.48–82.64%), while cefotaxime (40.83%; 95% CI: 34.31–47.35%), amoxicillin + clavulanic acid (33.49%; 27.22–39.76%), and imipenem (32.57%; 95% CI: 26.35–38.79%) demonstrated relevant intermediate levels of resistance. Fifty-six (25.68%; 95% CI: 19.88–31.48%) strains were resistant to the penicillins class, 18 (8.25%; 95% CI: 4.60–11.90%) to the cephalosporins class, and 29 (13.30%: 95% CI: 8.79–17.81%) strains were found resistant to both of them, for a total of 103 (47.24%; 95% CI: 40.61–53.87%) strains; while 37 (16.97%; 95% CI: 11.99–21.95%) strains were resistant to the carbapenems class. The results of the agar disk diffusion test are reported in Figure 1 and Table S1.

In relation to the results of the disk diffusion method, it was found that 150 (68.81%; 95% CI: 62.66–74.96%) strains did not fall into any of the resistance classes; among them, 91 (60.66%; 95% CI: 52.84–68.48%) were susceptible to all antimicrobials, 43 (28.66%; 95% CI: 21.42–35.90%) were resistant to at least one antimicrobial of one category, while 16 (10.66%; 95% CI: 5.72–15.60%) were resistant to at least one antimicrobial of two categories (Table 3).

Sixty-two (28.44%; 95% CI: 22.45–34.43%) strains were classified as MDR and 6 (2.75%; 95% CI: 0.58–4.92%) as XDR. Table 4 and Table 5 report the antimicrobial patterns for the MDR and XDR strains, respectively.

### 3.3. Genotypic Resistance

Molecular analyses conducted on strains phenotypically resistant to carbapenems, cephalosporins, and penicillins found 9/37 (24.32%; 95% CI: 10.50–38.14%) strains positive for *bla*_OXA-48_, 52/103 (50.49%; 95% CI: 40.83–60.15%) for *bla*_TEM,_ 12/103 (11.65%; 95% CI: 5.45–17.85%) for *bla*_CMY2_, 3/103 (2.91%; 95% CI: 0.00–6.16%) for *bla*_CTX_, and 1/103 (0.97%; 95% CI: 0.00–2.86%) for *bla*_SHV_ (Table 6). On the basis of these results, 9 isolates having only the *bla*_CMY2_ gene were identified as AmpC β-lactamases producers; 9 with only the *bla*_OXA-48_ gene were classified as carbapenemases producers; and 54 isolates having *bla*_TEM_ and/or *bla*_CTX_ and or *bla_SHV_* alone or in combination were identified as ESBLs.

Sequencing analyses of the amplicons showed 100% homology with the correspondent sequence reported in GenBank and therefore confirmed the positive results.

### 3.4. Virulence Factors

Molecular analyses to detect genes coding for the virulence factors characterizing different pathotypes found the *ast*A gene in 40/218 (18.35%; 95% CI:13.21–23.49%) isolates, potentially indicative of the EAEC pathotype. Additionally, 4/218 (1.83%; 95% CI: 0.05–3.61%) isolates had both *eae*A and *esc*V, potentially placing the strains within the EPEC pathotype. Moreover, 2/218 (0.92%; 95% CI: 0.00–2.19%) isolates tested positive for *cnf*1, 2/218 (0.92%; 95% CI: 0.00–2.19%) for *cnf*2, and 1/218 (0.46%; 95% CI: 0.00–1.36%) for *cdt*-IV, for a total of 5 (2.29%; 95% CI: 0.30–4.28%) potential NTEC strains. Among the strains classified as belonging to the investigated pathotypes, only 6 EAEC isolates were MDR.

Sequencing analyses of the amplicons showed 100% homology with the correspondent sequence reported in GenBank and therefore confirmed the positive results.

## 4. Discussion

The results obtained in the present survey show that seagulls may disperse through their droppings *E. coli* strains reportable as belonging to different pathotypes able to determine disease both in animals and humans. Seagulls fly long distances, reaching not only coastal zones, but also urban, peri-urban, and farm areas where they find human and animal waste from which they may acquire different pathogens. On the other hand, they can transport these microorganisms to additional areas, including beaches, acting as sources of infection.

*E. coli* is known to be ubiquitous and it has been frequently found in seabirds. However, few studies have investigated the occurrence of *E. coli* pathotypes in avifauna including gulls. Recently, Cardoso et al. [32], in Brazil, detected virulence genes of the EAEC, ETEC, or EPEC pathotypes in 30% of the identified strains, the first two described in seabirds for the first time. EPEC were previously reported in seagulls and other seabirds in the USA [33], and in wild birds, but not gulls, in Japan [34]. A survey carried out in wild birds of different species sampled in Italy evaluated the presence of virulence genes directly in birds’ feces; one or more virulence genes belonging to EPEC, EHEC, and STEC were found in 21/121 birds; 3 and 5 yellow-legged seagulls (*L. michaehellis*) had *stx1* and *eaeA* genes, respectively [35]. Sanches et al. [2] in Brazil isolated 401 *E. coli* strains from 516 wild birds and molecular analyses detected EPEC (2.99%) and STEC (0.74%). Similarly, Borges et al. [36] identified STEC (0.8%) and EPEC (2.0%) in feces of 123 free living wild birds from Brazil. No strains isolated in our survey were positive for *stx1* and/or *stx2* genes, suggesting that the examined gulls were not shedders of STEC strains. This result is in agreement with previous studies that did not detect STEC in feces sampled from gulls and other wild birds [34,37]. Conversely, other surveys found STEC in feces of wild birds belonging to different species, although with low rates [2,35,36,38,39,40,41,42]. Among the isolates of our study, 18.35% had the *astA* gene reportable as an EAEC strain, and 1.83% had the *eaeA* and *escV* genes typical of EPEC. These findings confirm wild avifauna, in particular seagulls, as possible spreaders of EAEC and EPEC.

All these pathotypes are relevant in human medicine. EPEC causes the loss of intestinal microvillus and induces a high child mortality rate, mainly in developing countries [42]. The EAEC pathotype is known as a major cause of acute and persistent diarrhea, and death among children in developing countries, but it is a cause of sporadic diarrhea and a common cause of traveler’s diarrhea, as well [43].

A relevant result obtained in the present study is the 2.29% (5/218) of strains with *cnf1*, *cnf2*, or *cdt*-IV genes coding for the typical virulence factors of the NTEC pathotype. To the best of our knowledge, data about the occurrence of NTEC in wild avifauna are not available in literature; therefore, this is the first report suggesting that gulls can harbor this pathotype, as well. NTEC strains were reported for the first time in neonatal enteritis [44]; in humans, these are associated with dysenteric syndrome, but it is also frequently involved in extra-intestinal infections, such as urinary tract infections [45]. Some investigations reported the presence of NTEC in mammals: NTEC1 strains have been isolated from ruminants, pigs, horses, dogs, and rabbits with enteritis and from pigs, dogs [46], and cats [47] with extra-intestinal infections; NTEC2 strains have been mainly cultured from ruminants with septicemia or intestinal infections [48,49,50,51,52].

Although the antimicrobial resistance rates were not very high, our study provides evidence that seagulls may contribute to the dissemination of *E. coli* strains characterized by resistance to different antimicrobials. The highest percentages of strains were resistant to ampicillin (38.99%), tetracycline (23.85%) and enrofloxacin (21.10%). These values are quite in accordance with the results of other surveys that evaluated the antimicrobial resistance of *E. coli* isolated from seagulls’ feces. Ahmed et al. [21] found 28%, 32%, and 24% of isolates were resistant to ampicillin, tetracyclines, and enrofloxacin, respectively, in Turkey. In Alaska, 27% of *E. coli* isolates were resistant to ampicillin and 44% to tetracyclines [53]. Moreover, our results are in line with research conducted on seagulls in other European areas. In a study conducted in the Czech Republic, 19.1% (49/257) of a total of 257 *E. coli* strains were found to be resistant to tetracycline and 11.7% (30/257) to ampicillin [54]. Resistance rates of 35% for tetracycline and 34% for amoxicillin were found in *E. coli* isolates from 179 seagull fecal samples in Portugal [55].

High percentages of resistance were found for ampicillin (72.22%), tetracycline (44.44%), and enrofloxacin (38.88%) in *E. coli* isolated from storks and seagulls in Central Spain [18]. In addition, Zendri et al. [56] found 100% resistance to ampicillin and 56.8% to tetracycline in *E. coli* from seagull feces in the UK. Furthermore, a survey conducted on *E. coli* isolated from seagull feces collected from nine different European countries (Denmark, England, Ireland, Latvia, Netherland, Poland, Portugal, Spain, Sweden) identified overall prevalence rates of 19.0% and 18.01% resistance to tetracycline and ampicillin, respectively [57]. Ampicillin, tetracycline, and enrofloxacin are among the most widely used antibiotics in both human and veterinary medicine in Europe, including Italy [58,59]. Ampicillin is commonly prescribed in human medicine for common bacterial infections, while tetracycline and enrofloxacin are widely used in veterinary medicine to treat bacterial infections in companion and farm animals [60,61].

Molecular analyses of our study revealed a high prevalence of the *bla_TEM_* gene (50.49%) in agreement with previous surveys. Dolejska et al. [54] found this gene in 29 of 30 *E. coli* isolates resistant to beta-lactam antibiotics in the Czech Republic. Poeta et al. [62] found 8/11 strains positive for *bla_TEM-52_* gene in Berlengas Island (Portugal) and, in the same area, Alves et al. [55] found that *bla*_TEM_ was the most prevalent gene in isolates from seagull feces (38% of 68 isolates resistant to penicillins). This gene encodes the TEM β-lactamase. This enzyme was originally linked to penicillin resistance, but over time, its variants have evolved into extended spectrum β-lactamases (ESBLs), enabling them to evade a broader range of β-lactam antibiotics, including cephalosporins and monobactams [63,64].

The *bla_CTX_* gene was found at a lower percentage (2.91%) than in *E. coli* from seagull feces from Porto (Portugal) beaches [20], where 98% of the 45 ESBL producers carried the *bla_CTX-M_* gene, and lower than the 41.6% (30/72) of *E. coli* isolated from gulls in Barcelona (Spain) [65]. Zendri et al. [56] found the *bla_CTX-M_* gene in 21/60 (35%) of the extended-spectrum cephalosporin-resistant *E. coli* strains isolated from seagulls in the UK. The *bla_CTX_* gene is responsible for the production of CTX-M β-lactamase, an ESBL particularly effective against cephalosporins, such as cefotaxime, contributing to the widespread antibiotic resistance in clinical bacteria [63].

Regarding the *bla_CMY-2_* gene, this is the most common plasmid-mediated AmpC β-lactamase in *Enterobacterales* and confers resistance to cephalosporins, penicillins, and combinations of antibiotics with β-lactamase inhibitors [66,67]. In our study, it was detected in 11.65% (12/103) of the analyzed strains, while Vergara et al. [65] found it in 2.8% (2/72) of *E. coli* strains isolated from gulls in Spain. Poirel et al. [68] reported a high percentage (29%) of *bla_CMY-2_* positive strains among resistant *E. coli* isolated from wild seagull feces in Miami Beach (USA).

Only one (0.97%, 1/103) of our strains was found positive for *bla*_SHV_, less than the 52.8% (38/72) detected in Spain [66]. The *bla*_SHV_ gene encodes SHV β-lactamase, an ESBL that destroys penicillins and broad-spectrum cephalosporins, commonly found in nosocomial infections [63]. SHV β-lactamases currently encompass a large number of allelic variants including extended-spectrum β-lactamases (ESBL), which are the majority, non-ESBL strains and several not classified variants [69].

The *bla*_OXA-48_ gene was found in 24.32% (9/37) of the carbapenems-resistant strains. Poeta et al. [62] found 1/11 resistant strain having the *bla*_OXA-1_ gene from seagulls of Berlengas Island. In contrast, Alves et al. [55] and Dolejska et al. [54] found no strain having the *bla*_OXA_ gene among 157 and 30 resistant *E. coli* isolates studied in Portugal and the Czech Republic, respectively. The *bla*_OXA-48_ gene encodes the enzyme OXA-48, a carbapenemase that makes bacteria resistant to carbapenems, which are used as antibiotics of last resort. Carbapenems are used as last-resort antibiotics to treat severe infections caused by MDR bacteria when other treatments have failed, owing to their limited vulnerability to most beta-lactam resistance determinants [68,70].

Finally, analyses of the distribution of resistance classes identified 28.44% of isolates as MDR and 2.75% as XDR. These results are similar to those found in other seagull populations, where the prevalence of MDR strains was 29% [21]. Similar values were also found by Martín-Maldonado et al. [18], who detected 63.2% (12/19) of the tested seagulls had antimicrobial-resistant *E. coli* strains, and four (30%) of them were considered MDR.

Our study could have some limitations. The first one concerns the analyzed samples. In fact, the number of fecal specimens was not high; however, only seagulls that had not received any antimicrobial treatment and had been recovered for no more than 48 h were selected. A further limit could be related to the health status of the gulls involved in the study; birds brought to rehabilitation centers usually are not in good health and therefore they are more susceptible to acquiring pathogens; however, the short time between the birds arriving and the sampling time should reduce this risk. A concern could also regard the antimicrobial susceptibility results, which might not reflect the real scenario, because the Kirby–Bauer method might be less sensitive than the broth microdilution test. In addition, molecular analyses to detect other antimicrobial resistance genes may be useful to better understand the role of *E. coli* strains as potential donors of these genes. These analyses should include the genes related to the detected resistance, as well as genes that may not been expressed.

## 5. Conclusions

The results obtained in this study showed that seagulls often harbor *E. coli* strains belonging to pathotypes responsible for diseases in humans and animals. Yellow-legged seagulls seem to be also involved in the epidemiology of the NTEC strains, which had never been detected in wild birds. Moreover, many *E. coli* strains isolated in this study were MDR and XDR and other isolates, even if not classified in these groups, were characterized by multiple antimicrobial resistances. The finding of resistance genes highlighted the additional issue related to the possibility that *E. coli* strains act as donors of these genes to other bacteria, contributing to the amplification of the antimicrobial resistance. Considering that seagulls are free-living wild birds not submitted to antimicrobial treatments, the detection of antimicrobial-resistant bacteria, including important pathogens, shows that they easily acquire bacteria from contaminated environments. Similarly, they can significantly contribute to the dissemination of pathogenic antimicrobial-resistant bacteria.

Therefore, targeted interventions, including improved waste management practices and public awareness campaigns, are essential to reduce seagulls’ access to areas where humans and other animals reside.

Seagulls could be used as effective indicators for monitoring the dissemination of different *E. coli* pathotypes and for studying old and new antimicrobial-resistances.

## Figures and Tables

**Table 1:** Primers and PCR conditions for the detection of the investigated resistance genes.

Target Gene	Primers Sequence (5′-3′)	Annealing Temp. (°C)	Amplicons Size (bp)	References
*bla*_NDM_	GGTTTGGCGATCTGGTTTTC CGGAATGGCTCATCACGATC	52	621	[27]
*bla*_KPC_	CGTCTAGTTCTGCTGTCTTG CTTGTCATCCTTGTTAGGCG	52	798	[27]
*bla*_OXA-48_	GCGTGGTTAAGGATGAACAC CATCAAGTTCAACCCAACCG	52	438	[27]
*bla*_IMP_	GGAATAGAGTGGCTTAAYTCTC GGTTTAAYAAAACAACCACC	52	232	[27]
*bla_VIM_*	GATGGTGTTTGGTCGCATA CGAATGCGCAGCACCAG	52	390	[27]
*bla*_TEM_	GCACGAGTGGGTTACATCGA GGTCCTCCGATCGTTGTCAG	60	310	[28]
*bla_SHV_*	TTCGCCTGTGTATTATCTCCCTG TTAGCGTTGCCAGTGYTCG	50	854	[29]
*bla_CTX-M_*	ATGTGCAGYACCAGTAARGTKATGGC TGGGTRAARTARGTSACCAGAAYCAGCGG	60	593	[29]
*bla_CMY-1_*	GTGGTGGATGCCAGCATCC GGTCGAGCCGGTCTTGTTGAA	58	915	[29]
*bla_CMY-2_*	GCACTTAGCCACCTATACGGCAG GCTTTTCAAGAATGCGCCAGG	58	758	[29]

**Table 2:** Primers and PCR conditions for the detection of virulence genes.

Pathotype	Target Gene	Primers Sequence (5′-3′)	Annealing Temp. (°C)	Amplicon Size (bp)	Reference
STEC/ EHEC	*stx1*	ATAAATCGCCATTCGTTGACTAC AGAACGCCCACTGAGATCATC	60	180	[30]
	*stx2*	GGCACTGTCTGAAACTGCTCC TCGCCAGTTATCTGACATTCTG	60	255	[30]
	*hylA*	GCATCATCAAGCGTACGTTCC AATGAGCCAAGCTGGTTAAGCT	60	534	[30]
EHEC/ EPEC	*eaeA*	GACCCGGCACAAGCATAAGC CCACCTGCAGCAACAAGAGG	60	384	[30]
EPEC	*escV*	ATTCTGGCTCTCTTCTTCTTTATGGCTG CGTCCCCTTTTACAAACTTCATCGC	53	544	[4]
	*bfpB*	GACACCTCATTGCTGAAGTCG CCAGAACACCTCCGTTATGC	53	910	[4]
	*ent*	TGGGCTAAAAGAAGACACACTG CAAGCATCCTGATTATCTCACC	53	629	[4]
ETEC	*LT*	GAACAGGAGGTTTCTGCGTTAGGTG CTTTCAATGGCTTTTTTTTGGGAGTC	53	655	[4]
	*STIa*	CCTCTTTTAGYCAGACARCTGAATCASTTG CAGGCAGGATTACAACAAAGTTCACAG	53	157	[4]
	*STI*	TGTCTTTTTCACCTTTCGCTC CGGTACAAGCAGGATTACAACAC	53	171	[4]
EIEC	*invE*	CGATAGATGGCGAGAAATTATATCCCG CGATCAAGAATCCCTAACAGAAGAATCAC	53	766	[4]
EAEC	*astA*	TGCCATCAACACAGTATATCCG ACGGCTTTGTAGTCCTTCCAT	53	102	[4]
	*aggR*	ACGCAGAGTTGCCTGATAAAG AATACAGAATCGTCAGCATCAGC	53	400	[4]
	*pic*	AGCCGTTTCCGCAGAAGCC AAATGTCAGTGAACCGACGATTGG	53	1111	[4]
NTEC	*CNF*1	GGGGGAAGTACAGAAGAATTA TTGCCGTCCACTCTCTCACCAGT	55	1111	[31]
	*CNF*2	TATCATACGGCAGGAGGAAGCACC GTCACAATAGACAATAATTTTCCG	55	1240	[31]
	*cdt-I*	CAATAGTCGCCCACAGGA ATAATCAAGAACACCACCAC	56	411	[31]
	*cdt-II*	GAAAATAAATGGAATATAAATGTCCG TTTGTGTTGCCGCCGCTGGTGAAA	56	556	[31]
	*cdt-III*	GAAAATAAATGGAATATAAATGTCCG TTTGTGTCGGTGCAGCAGGGAAAA	56	555	[31]
	*cdt-IV*	CCTGATGGTTCAGGAGGCTGGTTC TTGCTCCAGAATCTATACCT	56	350	[31]

**Table 3:** Antimicrobial patterns found with the 150 strains not falling into any of the resistance classes.

Antimicrobial Pattern	N. of Strains
Susceptible to all antimicrobials	91
CTX	12
AMP	8
IMP	8
ENR	4
AMP, AMC	3
AK	2
ETP	2
SXT	2
IMP, ETP	1
TE	1
AMP, AMC, FOX, CTX	2
AMP, ENR	2
AMP, ENR, CIP	2
AMP, TE	2
AMP, AMC, ETP	1
AMP, CTX	1
AMP, CTX, EFT	1
AMP, ETP	1
AMP, IMP	1
CTX, ENR, CIP	1
CTX, IMP, ETP	1
TE, ENR, CIP	1

Legend. AMP: ampicillin; AMC: amoxicillin-clavulanate; AK: amikacin; CIP: ciprofloxacin; CTX: cefotaxime; EFT: ceftiofur; ENR: enrofloxacin; ETP: ertapenem; FOX: cefoxitin; IMP: imipenem; SXT: trimethoprim-sulfamethoxazole; TE: tetracycline.

**Table 4:** Antimicrobial patterns found among the 62 MDR strains.

Antimicrobial Pattern	N. of Strains
AMP, AMC, CTX, EFT, ETP, ATM, ENR, CIP, AK	1
AMP, AMC, CTX, IMP, ETP, ATM, ENR, AK	1
AMP, AMC, CTX, IMP, C, TE, ENR, CIP	1
AMP, CTX, EFT, IMP, ETP, ATM, ENR, CIP, AK	1
AMP, CTX, IMP, C, TE, SXT	1
AMC, FOX, CTX, EFT, TE, ENR, SXT	1
AMP, AMC, C, TE, ENR, CIP, SXT	4
AMP, AMC, CTX, EFT, ATM, C, TE	1
AMP, AMC, ETP, C, ENR, CIP, SXT	2
AMP, AMC, FOX, CTX, TE, AK, SXT	1
AMP, C, TE, ENR, CIP, SXT	2
AMP, C, TE, ENR, SXT	1
C, TE, ENR, CIP, CN, SXT	3
AMP, AMC, C, ENR, CIP, SXT	1
AMP, AMC, C, TE, ENR, CIP	1
AMP, AMC, C, TE, SXT	1
AMP, AMC, CTX, EFT, IMP, SXT	1
AMP, AMC, CTX, EFT, TE, SXT	1
AMP, AMC, CTX, IMP, ENR	1
AMP, AMC, FOX, CTX, EFT, ATM, TE	1
AMP, AMC, TE, ENR, CIP, SXT	2
AMP, C, TE, ENR	1
AMP, C, TE, ENR, CIP	4 *
AMP, C, TE, SXT	6 *
AMP, CTX, EFT, IMP, AK	2 **
AMP, CTX, IMP, AK	1
FOX, CTX, EFT, ETP, ATM, CN, AK	1
FOX, CTX, ETP, ATM, CN, AK	1
FOX, ETP, ATM, CN, AK	1
AMP, AMC, CN, SXT	1
AMP, AMC, FOX, SXT	1 **
AMP, AMC, TE, ENR, CIP	1
AMP, C, TE	4
AMP, CTX, ETP	2
AMP, CTX, TE	1
AMP, ENR, CIP, SXT	1
AMP, TE, ENR	1
AMP, TE, SXT	3
FOX, CTX, IMP, ETP, AK	1

Legend. AMP: ampicillin; AMC: amoxicillin-clavulanate; AK: amikacin; ATM: aztreonam; C: chloramphenicol; CIP: ciprofloxacin; CN: gentamicin; CTX: cefotaxime; EFT: ceftiofur; ENR: enrofloxacin; ETP: ertapenem; FOX: cefoxitin; IMP: imipenem; SXT: trimethoprim-sulfamethoxazole; TE: tetracycline; *: 2 EAEC; **: 1 EAEC.

**Table 5:** Antimicrobial patterns found among the 6 XDR strains.

Antimicrobial Pattern	N. of Strains
AMP, FOX, CTX, EFT, ETP, ATM, C, TE, ENR, CIP, CN, AK, SXT	1
AMP, FOX, CTX, IMP, ETP, C, TE, ENR, CIP, AK, SXT	1
AMP, AMC, FOX, C, TE, ENR, CIP, CN, SXT	1
AMP, AMC, FOX, CTX, ETP, C, TE, ENR, CIP, SXT	1
AMP, CTX, EFT, C, TE, ENR, CIP, CN, SXT	2

Legend. AMP: ampicillin; AMC: amoxicillin-clavulanate; AK: amikacin; ATM: aztreonam; C: chloramphenicol; CIP: ciprofloxacin; CN: gentamicin; CTX: cefotaxime; EFT: ceftiofur; ENR: enrofloxacin; ETP: ertapenem; FOX: cefoxitin; IMP: imipenem; SXT: trimethoprim-sulfamethoxazole; TE: tetracycline.

**Table 6:** Antimicrobial resistance genes in *Escherichia coli* isolates from seagulls.

	Genes									
	*bla*_NDM_	*bla*_KPC_	*bla*_OXA-48_	*bla*_VIM_	*bla*_IMP_	*bla*_CMY1_	*bla*_CMY2_	*bla*_SHV_	*bla*_CTX_	*bla*_TEM_
No. positive isolates	0	0	9 (24.32%)	0	0	0	12 (11.65%)	1 (0.97%)	3 (2.91%)	52 (50.49%)
No. tested isolates	37	37	37	37	37	103	103	103	103	103

## Data Availability

All data are reported in the manuscript.

## Supplementary Materials

The following supporting information can be downloaded at https://www.mdpi.com/article/10.3390/ani14213048/s1, Table S1: Antimicrobial resistance profile of *Escherichia coli* isolates (n.218) from seagulls.
