# Supplementary material for: Antimicrobial Resistance and Pathotypes of Escherichia coli Isolates from Yellow-Legged Seagulls (Larus michahellis) in Central Italy
Source: Animals (Basel). 2024 Oct 22;14(21):3048. doi: 10.3390/ani14213048 (PMC11545632; doi:10.3390/ani14213048)
Supplement: Supplementary file 1 [file animals-14-03048-s001.zip › animals-3240053-supplementary.pdf]

Table S1. Antimicrobial resistance profile of *Escherichia coli* isolates (n.218) from seagulls.

Categories	Antibiotic	Susceptible		Intermediate		Resistant	
		N. of isolates	%	N. of isolates	%	N. of isolates	%
Penicillins	ampicillin	95	43.58	38	17.43	85	38.99
	amoxicillin-clavulanate	114	52.29	73	33.49	31	14.22
Cephalosporins	cefoxitin	168	77.06	36	16.51	14	6.42
	cefotaxime	85	38.99	89	40.83	44	20.18
	ceftiofur	169	77.52	35	16.06	14	6.42
Carbapenems	imipenem	124	56.88	71	32.57	23	10.55
	ertapenem	173	79.36	25	11.47	20	9.17
Monobactams	aztreonam	197	90.37	12	5.50	9	4.13
Phenicols	chloramphenicol	174	79.82	5	2.29	39	17.89
Tetracyclines	tetracycline	155	71.10	11	5.05	52	23.85
Fluoroquinolones	enrofloxacin	116	53.21	56	25.69	46	21.10
	ciprofloxacin	145	66.51	39	17.89	34	15.60
Aminoglycosides	gentamicin	198	90.83	9	4.13	11	5.05
	amikacin	162	74.31	41	18.81	15	6.88
Folate pathway antagonists	trimethoprim-sulfamethoxazole	165	75.69	12	5.50	41	18.81
